# Survival and Failure Outcomes Predicted by Brain Metastasis Volumetric Kinetics in Melanoma Patients Following Upfront Treatment with Stereotactic Radiosurgery Alone

**DOI:** 10.7759/cureus.1934

**Published:** 2017-12-11

**Authors:** Michael C LeCompte, Emory McTyre, Adrianna Henson, Michael Farris, Catherine Okoukoni, Christina K Cramer, Pierre Triozzi, Jimmy Ruiz, Kounosuke Watabe, Hui-Wen Lo, Michael T Munley, Adrian W Laxton, Stephen B Tatter, Xiaobo Zhou, Michael Chan

**Affiliations:** 1 Department of Radiation Oncology, Wake Forest School of Medicine; 2 Department of Medicine (hematology & Oncology), Wake Forest School of Medicine; 3 Department of Cancer Biology, Wake Forest School of Medicine; 4 Department of Neurosurgery, Wake Forest School of Medicine; 5 Center for Bioinformatics & Systems Biology, Wake Forest School of Medicine; 6 Department of Radiation Oncology, Wake Forest University

**Keywords:** brain metastasis, brain metastasis velocity, melanoma, radiosurgery, whole brain radiotherapy

## Abstract

Introduction

The roles of early whole brain radiotherapy (WBRT) and upfront stereotactic radiosurgery (SRS) alone in the treatment of melanoma patients with brain metastasis remain uncertain. We investigated the volumetric kinetics of brain metastasis development and associations with clinical outcomes for melanoma patients who received upfront SRS alone.

Methods

Volumetric brain metastasis velocity (vBMV) was defined as the volume of new intracranial disease at the time of distant brain failure (DBF) for the first DBF (DBF1) and second DBF (DBF2) averaged over the time since initial or most recent SRS. Non-volumetric brain metastasis velocity (BMV) was calculated for comparison.

Results

Median overall survival (OS) for all patients was 7.7 months. Increasing vBMV_DBF1_ was associated with worsened OS (hazard ratio (HR): 1.10, confidence interval (CI): 1.02 - 1.18, p = .01). Non-volumetric BMV_DBF1_ was not predictive of OS after DBF1 (HR: 1.00, CI: 0.97 - 1.02, p = .77). Cumulative incidence of DBF2 at three months after DBF1 was 50.0% for vBMV_DBF1_ > 4 cc/yr versus (vs) 15.1% for vBMV_DBF1_ ≤ 4 cc/yr, (Gray’s p-value = .02). Cumulative incidence of salvage WBRT at three months after DBF1 was 50.0% for vBMV_DBF1_ > 4 cc/yr vs 2.3% for vBMV_DBF1_ ≤ 4 cc/yr (Gray’s p-value < .001).

Conclusion

In melanoma patients with brain metastasis, volumetric BMV was predictive of survival, shorter time to second DBF, and the need for salvage WBRT. Non-volumetric BMV, however, did not predict for these outcomes, suggesting that vBMV is a stronger predictor in melanoma.

## Introduction

Melanoma is among the most common histologies to metastasize to the brain [1-2]. Prognosis is generally poor, as the median survival for these patients once they have developed brain metastases has been reported to be from three to 13 months in spite of modern therapies [3-4]. The survival range is wide, however, and a minority of patients do survive more than three years [5]. Among the hallmarks of melanoma brain metastasis, biology is the propensity for re-seeding of the brain. The presence of brain metastasis has been found in multiple series to be an independent predictor of intracranial progression [6-7].

The use of whole brain radiotherapy (WBRT) and its optimal timing is particularly controversial in the melanoma brain metastasis population. The theoretical advantage of WBRT is its ability to treat occult brain metastases and thus mitigate distant brain failure [6, 8]. However, WBRT has cognitive toxicities and may affect performance status [9]. Alternatively, stereotactic radiosurgery (SRS) has been used to avoid the toxicities of WBRT, though the distant brain failure risk is much higher with SRS alone. Identifying the proper time to use WBRT during a patient’s disease course with melanoma is a challenge that has yet to be fully addressed.

The use of SRS as a single modality is expanding, and recent series suggests that treating as many as 10 lesions with SRS alone may be beneficial [10-11]. Moreover, modern studies suggest that the cumulative tumor volume may be more predictive of survival than the absolute number of metastases [11-12]. A recent series has introduced brain metastasis velocity (BMV) as a novel clinical metric that models the rate of development of new brain metastases after initial SRS and found this to be predictive of both survival and the need for WBRT salvage [13]. As melanoma patients are purported to have higher BMV than other histologies, we performed a study to assess the prognostic significance of the velocity of cumulative tumor volume or vBMV in melanoma patients with brain metastases, while comparing its prognostic value to the value of numerical BMV in the same population.

## Materials and methods

Data acquisition

This study was approved by the Institutional Review Board at Wake Forest Baptist Medical Center. The IRB allowed waiver of consent, given the retrospective nature of the analysis of pre-existing data and therefore minimal risk to patients. Data were reviewed and collected for patients treated between January 2000 and December 2015 at our institution. The current series is an extension of a previously published study [13]. Patients were included in the study if they underwent SRS alone with no initial WBRT following initial diagnosis with brain metastasis. A total of 107 patients were identified that met the criteria. Fifty-four of these patients were included in the previously published study [13]. Electronic medical records were reviewed to determine the clinicopathologic characteristics, which have previously been determined to contribute towards overall survival (OS) and distant brain failure (DBF) in brain metastasis patients [14]. The burden of extracranial disease was defined based on prior published works [15]. Radiosurgical doses were generally determined based upon the guidelines published by Shaw, et al. for single fraction radiosurgical treatment of brain metastases [16].

Response assessment and follow-up

Patients underwent follow-up magnetic resonance imaging (MRI) of the brain and had a clinical visit at four to eight weeks after the initial SRS procedure. Follow-up MRI was generally acquired using a slice thickness of 1.25 mm or 2.5 mm. The specifics of the protocol varied by the MRI unit with which the study was acquired. Subsequent MRI’s and clinical visits were done every three months for the first two years and then four to six months subsequently. Distant brain failures were determined to be any new metastasis that developed outside of the SRS treatment volume.

For volumetric analysis, follow-up MRI Digital Imaging and Communications in Medicine (DICOM) files associated with each DBF were imported into the GammaPlan Treatment Planning System (Elekta AB, Stockholm, Sweden). The volumes of each brain metastasis at the time of DBF were then segmented in GammaPlan, and three-dimensional volume (mL) measurements were acquired.

Brain metastasis velocity

Volumetric BMV (vBMV) was defined by performing separate linear regressions for each patient for the cumulative volume of new brain metastases at each failure point versus (vs) time from initial SRS. The slope of the linear regression fit line was used to assign a vBMV value to each patient. vBMV was estimated for all patients with DBF with a quantifiable volume of brain metastases, which was performed over the interval from initial SRS until most recent imaging or until receiving WBRT, as the latter was considered to be a modifier of vBMV. Patients who had at least six months of follow-up imaging and had no DBF were assigned a vBMV of 0. There was no formal policy regarding salvage, however, our institutional practice was to offer SRS salvage to patients with DBF with a relatively small number of brain metastases, while patients with a larger burden of intracranial disease at the time of DBF were more frequently offered WBRT.

Although estimation of vBMV with linear regression was considered a more robust method, in order to maximize clinical applicability for patients who have already experienced DBF a simplified vBMV estimate was calculated:

$vBMV = \frac{Cumulative\: volume\: of\: new\: brain\: metastases\: since\: initial\: SRS}{Totat\: time\: (yrs)\: between\: initial\: SRS\: and\: Time\: (i)}$

vBMV_Time(i)_ was defined for multiple timepoints in this study, including time of first DBF (vBMV_DBF1_) and second DBF (vBMV_DBF2_).

Patients were classified by initial vBMV into low and high-risk groups, consisting respectively of vBMV_DBF1_ ≤ 4 cc/yr and > 4 cc/yr. Sorting into these groups offered the most significant threshold. Patients were then grouped by vBMV into low and high-risk groups based upon a clinical determination of the likelihood of salvage WBRT, the likelihood of a second DBF event, and death associated with given vBMV values.

Non-volumetric BMV was defined using similar parameters. A simplified non-volumetric BMV_regression_ estimate was calculated:

$BMV = \frac{Cumulative\: number\: of\: new\: brain\: metastases\: since\: initial\: SRS}{Totat\: time\: (yrs)\: between\: initial\: SRS\: and\: Time\: (i)}$

Patients were classified by the initial BMV into low and high-risk groups, consisting respectively of BMV ≤ 6 new metastases/yr and > 6 new metastases/yr. These groups were identified by Farris, et al. [13].

Radiosurgical technique

Patients were treated with the Leksell Gamma Knife B, C, or Perfexion units (Elekta AB, Stockholm). Prior to radiosurgical treatment, patients underwent a high-resolution stereotactic MRI of the brain. Treatment planning was performed on the Leksell GammaPlan Treatment Planning System (Elekta AB, Stockholm). A median dose of 19 Gy (interquartile range (IQR): 18 - 20 Gy) was prescribed to the 50% isodose line to each lesion according to the dose guidelines published by Shaw, et al. [16].

Statistical analysis

Median follow-up and time-to-event outcomes were defined as the time from SRS to the time of most recent follow-up or to the event of interest. Time-to-event outcomes were summarized using the Kaplan-Meier estimator with log-rank tests performed for stratified outcomes. Cumulative incidences were estimated for the second DBF and time to salvage WBRT following the initial DBF [17]. Competing risks models were developed to estimate the single variable subdistribution hazard ratios (HR) associated with each predictor for each of these events. Statistically significant (p < .05) variables identified on univariate analysis were included in forward stepwise regressions to identify the multivariable models that minimized the Akaike information criterion (AIC) [18]. Calculated p-values were from two-sided tests. These results were used to guide the purposeful development of multivariate competing risks models for DBF vs death prior to DBF, as well as first salvage with SRS vs first salvage with WBRT vs death prior to salvage/DBF.

Linear regression was performed for the predictor variable vBMV_DBF1_ to determine the kinetics of future development of intracranial disease for the outcome variables vBMV_DBF2_, time to DBF2, and total brain metastasis volume at the time of DBF2.

We report the current study in accordance with the Strengthening the Reporting of Observational Studies in Epidemiology (STROBE) guidelines.

## Results

Patient population

A total of 107 patients were identified who had melanoma with brain metastases initially treated with SRS alone. Forty-one patients (38.3%) had received prior targeted therapy with dabrafenib, vemurafenib, and/or trametinib, and 13 patients (12.1%) had received both immunotherapy and targeted therapy. The baseline characteristics of these patients can be found in Table 1. The median follow-up for all patients was 5.4 years.

**Table 1:** Patient Characteristics at the Time of Initial Stereotactic Radiosurgery (SRS) n: number; IQR: interquartile range; F: female; M: male; KPS: Karnofsky performance status; cc: cubic centimeters

	Overall
n	107
Age (median {IQR})	63.00 {53.00-72.25}
Sex (%)	
F	31 (29.0)
M	76 (71.0)
BRAF status (%)	
Wildtype	21 (19.6)
Mutated	20 (18.7)
Unknown	66 (61.7)
Extent of extracranial disease (%)	
None	32 (29.9)
Oligometastatic (≤ 5 extracranial mets)	56 (52.3)
Widespread (> 5 extracranial mets)	15 (14.0)
Unknown	4 (3.7)
Prior systemic therapy (%)	
Any targeted agent	41 (38.3)
Ipilimumab	33 (30.8)
Vemurafenib	10 (9.3)
Dabrafenib	8 (7.5)
Trametinib	7 (6.5)
Pembrolizumab	5 (4.7)
KPS (%)	
60	8 (7.5)
70	18 (16.8)
80	50 (46.7)
90	28 (26.2)
100	3 (2.8)
Initial number of brain metastases (%)	
1	47 (43.9)
2	23 (21.5)
3	17 (15.9)
4	8 (7.5)
5	2 (1.9)
≥ 6	10 (9.4)
Total volume of all brain metastases at initial SRS, cc (median {IQR})	2.81 {0.84-7.79}

Outcomes

Median OS for all patients was 7.7 (CI: 5.3 - 11.0) months. Actuarial OS at six, 12, and 24 months for all patients was 56.3%, 35.8%, and 21.6%, respectively. Seventy-four patients (69.2%) were documented with a DBF event following initial SRS, with the estimated cumulative incidence of DBF at six and 12 months being 57.1% and 64.1%, respectively. All patients assigned a vBMV_DBF1 _were documented with a DBF event. Eighteen patients assigned a vBMV_DBF1 _had a DBF2 event. A total of 27 patients (25.2%) received WBRT prior to death, with the estimated cumulative incidence of salvage WBRT at six and 12 months being 18.0% and 23.9%, respectively.

Outcomes as stratified by vBMV

**Figure 1:**
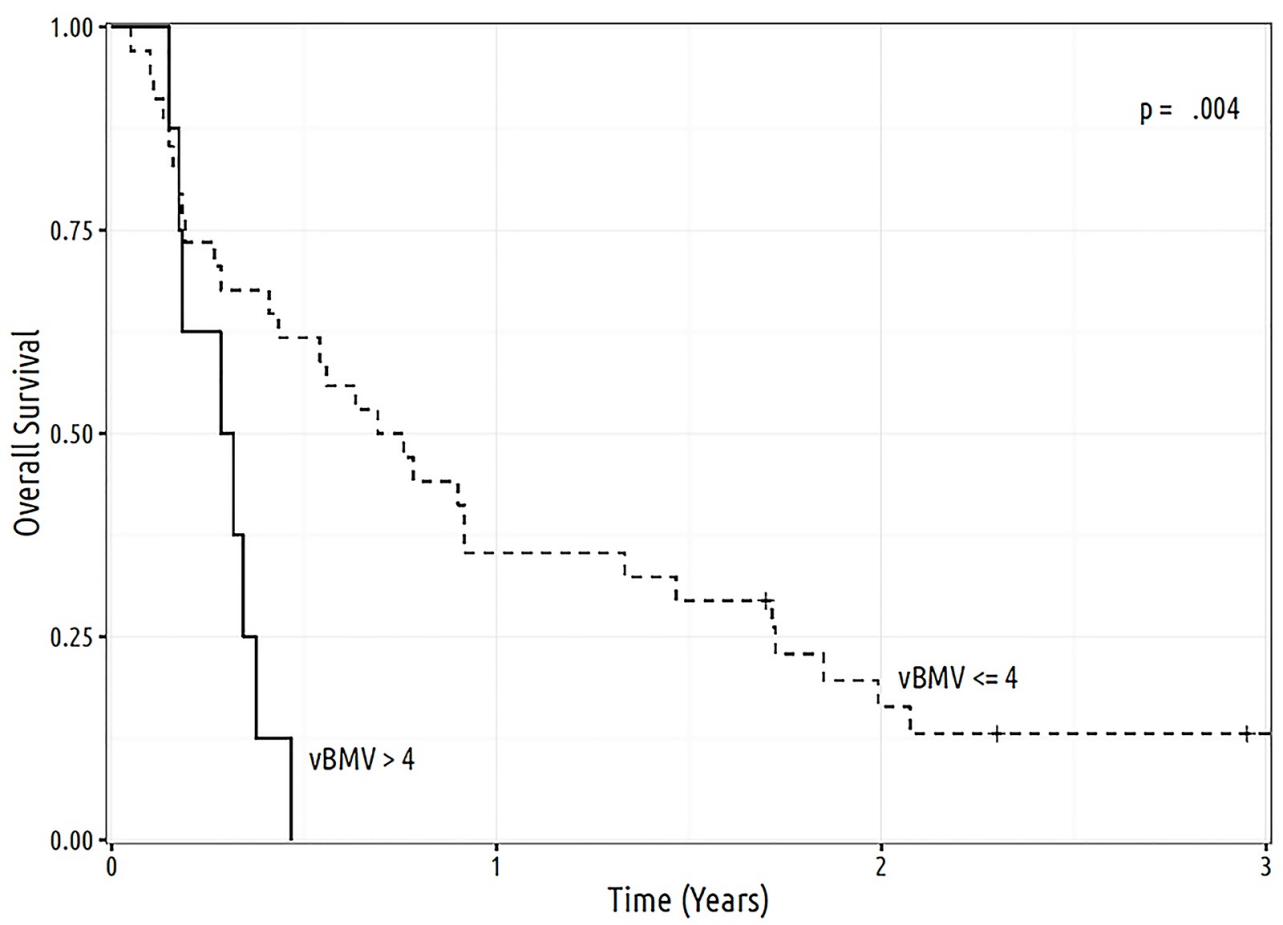
Overall survival according to vBMV as calculated at time of first distant brain failure and separated into low and high risk groups categories, consisting respectively of ≤ 4 cc/yr and > 4 cc/yr. cc: cubic centimeter; vBMV: volumetric brain metastasis velocity; yr: year

Brain metastasis kinetics analysis was performed for all the patients. The median vBMV_DBF1_ was 1.0 cc/yr (IQR: 0.3 - 3.2 cc/yr), while the median vBMV_DBF2_ was 1.3 cc/yr (IQR: 0.5 - 4.1 cc/yr). When stratifying by vBMV_DBF1_ ≤ 4 cc/yr vs 4 cc/yr, median OS after the initial DBF was 8.7 (CI: 5.2 - 17.6) months vs 3.6 (CI: 2.2 - not available (NA)) months, respectively (p = .004) (Figure 1). One-year OS for vBMV_DBF1_ ≤ 4 cc/yr vs > 4 cc/yr was 35.3% vs 0.0%, respectively. As a continuous variable, increasing vBMV_DBF1_ was associated with worsened OS (HR: 1.10, CI: 1.02 - 1.18, p = .01). Non-volumetric BMV_DBF1_ was not predictive of OS, either as a continuous variable (HR: 1.00, CI: 0.97 - 1.02, p = .77) or when categorized into ≤ 6 new metastases/yr vs > 6 new metastases/yr (HR: 1.38, CI: 0.72 - 2.66, p = .33).

Brain metastasis kinetics

**Figure 2:**
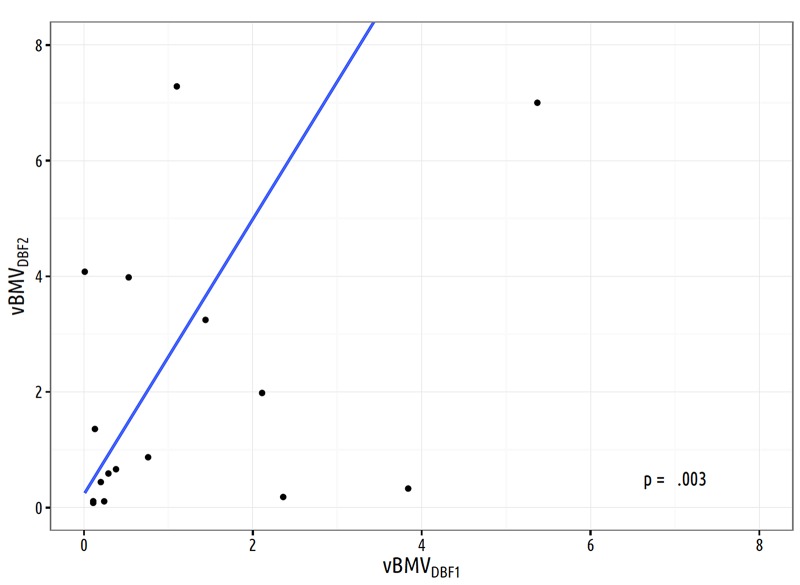
Linear regression analysis based on 18 patients for the volume of distant brain metastases velocity at the time of second distant brain failure vs vBMV calculated at time of first distant brain failure (β = 2.4, SE = 0.7). SE: standard error; vBMV: volumetric brain metastasis velocity; DBF1: first distant brain failure; DBF2: second distant brain failure

Linear regression for the predictor variable vBMV_DBF1_ for the outcome of vBMV_DBF2_ showed an association between these variables, with β = 2.4 (SE: 0.7, p = .003) (Figure 2). Cumulative incidence of DBF2 at three months after DBF1 was 50.0% for vBMV_DBF1_ > 4 cc/yr vs 15.1% for vBMV_DBF1_ ≤ 4 cc/yr, (Gray’s p-value = .02). Non-volumetric BMV_DBF1_ was not predictive of DBF2, either as a continuous variable (HR: 1.00, CI: 0.97 - 1.03, p = .88) or when categorized into ≤ 6 new metastases/yr vs > 6 new metastases/yr (HR: 1.54, CI: 0.67 - 3.58, p = .31). Cumulative incidence of salvage WBRT at three months after DBF1 was 50.0% for vBMV_DBF1_ > 4 cc/yr vs 2.3% for vBMV_DBF1_ ≤ 4 cc/yr, (Gray’s p-value < .001). Non-volumetric BMV_DBF1_ was not predictive of incidence of salvage WBRT, either as a continuous variable (HR: 0.98, CI: 0.95 - 1.01, p = .28) or when categorized into ≤ 6 new metastases/yr vs > 6 new metastases/yr (HR: 2.41, CI: 0.50 - 11.60, p = .27).

Linear regression found the presence of widespread extracranial disease to be predictive of higher vBMV_DBF1_ (β = 3.57, p = .042) (Table 2). Regression also indicated the inclusion of targeted therapy in treatment was predictive of lower vBMV_DBF1_ (β = -2.62, p = .038) (Table 2).

**Table 2:** Multiple Regression Model for Predictors of Volumetric Brain Metastasis Velocity at the Time of Initial Distant Brain Failure vBMV: volumetric brain metastasis velocity; DBF1: first distant brain failure; CI = confidence interval, adj. = adjusted

	vBMV_DBF1_		
	β	95% CI	p
(Intercept)	8.86	2.53 - 15.19	.007
Age	-0.08	-0.17 - 0.01	.077
Widespread extracranial disease	3.57	0.14 - 6.99	.042
Targeted therapy	-2.62	-5.08 - -0.16	.038
R^2^ / adj. R^2^	0.261 / 0.201		

## Discussion

In a recent series, our group defined BMV as a prognostic indicator for patients with brain metastases [13]. Lower BMV predicted an improved survival and was associated with decreased rates of salvage treatment to the brain and neurologic death. A limitation of the study by Farris, et al. was the reliance on the assumption that the number of new brain metastases is an accurate surrogate for the amount of new intracranial disease, particularly as studies have shown that the total volume of brain metastases may be more prognostic than merely the number of lesions alone [19]. In the current study, we aim to address this issue of the importance of volumetric vs. numerical (non-volumetric) BMV. We chose melanoma as a cohort of interest because melanoma histology was among the dominant factors associated with higher BMV in the previous study [13] and because there is a divergence in the possible clinical outcomes after SRS for melanoma patients.

The current series suggests that vBMV in comparison to numerical BMV is a stronger predictor of survival and shorter time to second DBF and WBRT salvage in melanoma patients with brain metastases initially treated with SRS alone. Previous studies identified higher cumulative tumor volume as a predictor for poorer OS [11-12]. In the current series, vBMV_DBF1 _is associated with a positive correlation with OS as both a continuous and a stratified variable. Numerical BMV is not predictive of OS after an initial DBF event, either as a continuous variable or when categorized into ≤ 6 new metastases/yr vs > 6 new metastases/yr.

Distant brain failure and brain metastasis kinetics

Rodrigues, et al. reported a smaller cumulative tumor volume to be predictive of increased rates of DBF [20]. In concordance, Press, et al. also found that the cumulative tumor volume of < 1.3 cc predicted increased rates of DBF [21]. Lower gross volume may be indicative of a biological phenotype with an ability to seed with multiple micrometastases as opposed to larger single lesions [21]. An alternative mechanism for DBF is that small lesions may potentially be missed on the radiosurgical planning MRI and appear as increased rates of DBF [20-21]. The advantage of vBMV as a model of brain metastasis behavior is that it can help to distinguish biological differences over time as patients with occult metastases that were not detected at the time of SRS but with no further failures would have a low vBMV, whereas patients with continued progression would have a higher vBMV. In the current series, higher vBMV following the initial DBF event (vBMV_DBF1_ > 4 cc/yr) is predictive of a time to the second DBF event. vBMV provides a model for cumulative tumor volume over time -- a notable advantage to past volumetric and non-volumetric predictors found in the literature. Additionally, an association between vBMV_DBF1_ and vBMV_DBF2_ (β = 2.4, SE: 0.7, p = .003) (Figure 2) suggests that over time there is an acceleration in the development of intracranial disease. As the vBMV at the first DBF event increases, the volume of brain metastases is more likely to be increased at the time of the second DBF event. This observation is based on results from a small subpopulation in the current series and needs further validation. Acceleration of tumor growth potentially contributes to the progression of intracranial disease and could indicate the need for salvage WBRT.

Whole brain radiation therapy

A recent meta-analysis has suggested that a subset of patients may even see an advantage in survival when treated initially with SRS in comparison to SRS, plus WBRT [22]. The ability to predict when salvage WBRT is needed gives the added benefit of distant control and postpones toxic effects associated with WBRT until this treatment is necessary. The current series reports that higher vBMV following the initial DBF event (vBMV_DBF1_ > 4 cc/yr) is predictive of the need for salvage WBRT. Numerical BMV is not predictive of the need for salvage WBRT in the current series. As such, not only does vBMV outperform numerical BMV with regards to survival, but it also is the superior biomarker in the prediction of the need for WBRT.

Concurrent therapy for melanoma brain metastases

Recent studies have tested the safety and benefits of immunotherapy agents given concurrently with SRS [23-24]. A retrospective study that evaluated melanoma patients treated with SRS for brain metastasis found the use of ipilimumab to be associated with improved survival [25]. In the current series, use of immunotherapy and targeted therapies is predictive for lower vBMV_DBF1_ (Table 2). The use of concurrent immunotherapy and SRS has been associated with a greater median percentage reduction in lesion size when compared to non-concurrent therapy [26]. Additionally, a transient increase in size may be observed when using concurrent therapy, which possibly represents active immunomodulation [24].

Limitations of the study

There are limitations of the current series. As a retrospective cohort, it is subject to patient selection bias. Furthermore, some patients with rapidly progressive disease end up getting supportive care alone without further imaging. As a result, there is likely a population of patients with progressive intracranial disease that were not imaged prior to their demise. Finally, the time period during which the patients in this series were treated saw an improvement in systemic therapies that has somewhat altered the patterns of failure of brain metastases over time [5]. The recent series by Farris, et al. showed that numerical BMV was independent of the time era as a predictor of survival [13]. In spite of its limitations, the current study is of a large and detailed dataset in which every detected brain metastasis of each patient treated over a 15-year span was documented and used for derivation of the current model.

## Conclusions

We found vBMV to be an effective predictor of poorer overall survival and shorter time to second DBF and need for salvage WBRT within melanoma patients treated with upfront SRS alone. Patients with higher vBMV_DBF1_ values can potentially benefit from earlier salvage WBRT. Our results indicate that the velocity of cumulative tumor volume may be a better predictor of clinical outcomes in comparison to numerical BMV. Volumetric brain metastasis kinetics was also able to project the acceleration of intracranial progression and, therefore, may be taken into consideration to better estimate the development of intracranial disease. However, a prospective study will be needed to validate the findings of the current study.

## References

[1] Gavrilovic IT, Posner JB (2005). Brain metastases epidemiology and pathophysiology. J Neurooncol.

[2] Davies MA, Liu P, McIntyre S (2011). Prognostic factors for survival in melanoma patients with brain metastases. Cancer.

[3] Sperduto PW, Kased N, Roberge D (2012). Summary report on the graded prognostic assessment: an accurate and facile diagnosis-specific tool to estimate survival for patients with brain metastases. J Clin Oncol.

[4] Goyal S, Silk AW, Tian S (2015). Clinical management of multiple melanoma brain metastases: a systematic review. JAMA Oncol.

[5] Johnson AG, Ruiz J, Hughes R (2015). Impact of systemic targeted agents on the clinical outcomes of patients with brain metastases. Oncotarget.

[6] Dyer MA, Arvold ND, Chen YH (2014). The role of whole brain radiation therapy in the management of melanoma brain metastases. Radiat Oncol.

[7] Neal MT, Chan MD, Lucas JT Jr (2014). Predictors of survival, neurologic death, local failure, and distant failure after Gamma Knife radiosurgery for melanoma brain metastases. World Neurosurg.

[8] Aoyama H, Tago M, Shirato H (2015). Stereotactic radiosurgery with or without whole-brain radiotherapy for brain metastases: secondary analysis of the JROSG 99-1 randomized clinical trial. JAMA Oncol.

[9] Greene-Schloesser D, Robbins ME, Peiffer AM (2012). Radiation-induced brain injury: A review. Front Oncol.

[10] Yamamoto M, Kawabe T, Sato Y, Higuchi Y, Nariai T, Watanabe S, Kasuya H (2014). Stereotactic radiosurgery for patients with multiple brain metastases: a case-matched study comparing treatment results for patients with 2-9 versus 10 or more tumors. J Neurosurg.

[11] Yamamoto M, Serizawa T, Shuto T (2014). Stereotactic radiosurgery for patients with multiple brain metastases (JLGK0901): a multi-institutional prospective observational study. Lancet Oncol.

[12] Liew DN, Kano H, Kondziolka D (2011). Outcome predictors of Gamma Knife surgery for melanoma brain metastases. Clinical article. J Neurosurg.

[13] Farris M, McTyre ER, Cramer CK (2017). Brain metastasis velocity: A novel prognostic metric predictive of overall survival and freedom from whole-brain radiation therapy after distant brain failure following upfront radiosurgery alone. Int J Radiat Oncol Biol Phys.

[14] Ayala-Peacock DN, Peiffer AM, Lucas JT (2014). A nomogram for predicting distant brain failure in patients treated with Gamma Knife stereotactic radiosurgery without whole brain radiotherapy. Neuro Oncol.

[15] Harris S, Chan MD, Lovato JF (2012). Gamma knife stereotactic radiosurgery as salvage therapy after failure of whole-brain radiotherapy in patients with small-cell lung cancer. Int J Radiat Oncol Biol Phys.

[16] Shaw E, Scott C, Souhami L (2000). Single dose radiosurgical treatment of recurrent previously irradiated primary brain tumors and brain metastases: final report of RTOG protocol 90-05. Int J Radiat Oncol Biol Phys.

[17] Fine JP, Gray RJ (1999). A proportional hazards model for the subdistribution of a competing risk. J Am Stat Assoc.

[18] Akaike H (1974). A new look at the statistical model identification. IEEE Trans Automat Contr.

[19] Baschnagel AM, Meyer KD, Chen PY (2013). Tumor volume as a predictor of survival and local control in patients with brain metastases treated with Gamma Knife surgery. J Neurosurg.

[20] Rodrigues G, Warner A, Zindler J (2014). A clinical nomogram and recursive partitioning analysis to determine the risk of regional failure after radiosurgery alone for brain metastases. Radiother Oncol.

[21] Press RH, Prabhu RS, Nickleach DC (2015). Novel risk stratification score for predicting early distant brain failure and salvage whole-brain radiotherapy after stereotactic radiosurgery for brain metastases. Cancer.

[22] Sahgal A, Aoyama H, Kocher M (2015). Phase 3 trials of stereotactic radiosurgery with or without whole-brain radiation therapy for 1 to 4 brain metastases: individual patient data meta-analysis. Int J Radiat Oncol Biol Phys.

[23] Gaudy-Marqueste C, Carron R, Delsanti C (2014). On demand Gamma-Knife strategy can be safely combined with BRAF inhibitors for the treatment of melanoma brain metastases. Ann Oncol.

[24] Kiess AP, Wolchok JD, Barker CA (2015). Stereotactic radiosurgery for melanoma brain metastases in patients receiving ipilimumab: safety profile and efficacy of combined treatment. Int J Radiat Oncol Biol Phys.

[25] Henson A, McTyre E, Ayala-Peacock DN, Triozzi P, Savage P, Laxton AW, Chan MD (2016). Outcomes for metastatic melanoma treated with stereotactic radiosurgery in the era of targeted systemic therapies. Int J Radiat Oncol Biol Phys.

[26] Qian JM, Yu JB, Kluger HM, Chiang VL (2016). Timing and type of immune checkpoint therapy affect the early radiographic response of melanoma brain metastases to stereotactic radiosurgery. Cancer.

